# The Impact of Exposure to War and War-Related Stressors on Subjective Age and Accelerated Aging:

**DOI:** 10.1093/geroni/igaf122.1491

**Published:** 2025-12-31

**Authors:** Anna Kornadt, Oksana Senyk, Ehud Bodner, Yuval Palgi, Amit Shrira, Lee Kimron Greenblatt, Kvitoslava Khorob

**Affiliations:** University of Luxembourg, Esch-sur-Alzette, Grevenmacher, Luxembourg; WSB Merito University in Gdansk, Gdansk, Pomorskie, Poland; Bar-Ilan University, Ramat Gan, HaMerkaz, Israel; University of Haifa, Haifa, HaZafon, Israel, Israel; Bar-Ilan University, Ramat Gan, HaMerkaz, Israel; Ariel University, Ariel, HaMerkaz, Israel; Ukrainian Catholic University, Lviv, L’vivs’ka Oblast’, Ukraine

## Abstract

Previous research shows that the experience of adversity and extreme life events can have an impact on the experience of aging (e.g., Avidor et al., 2016; Hoffman et al., 2022). Still, empirical evidence is sparse regarding the impact of war experiences, especially in short-term and daily contexts. We thus investigated the role of exposure to war-related events, stressors, and their experience on subjective age and subjective accelerated aging in two samples of older adults exposed to war. The first sample includes N = 71 adults aged 50-78 years from the Ukraine, who reported on their exposure to war-related events as well as their daily experience of aging, and daily war-related stressors over 14 days during the third year of the Russian invasion of Ukraine. Multilevel regression analyses found that there was no effect of daily war-related stressors on daily subjective age (b = .45, p = .14) or accelerated aging (b = .11, p = .08), but on the between-person level, more exposure to war-related events was associated with feeling older (b = 1.10, p <.001). We will expand these findings with a second sample of Israeli adults aged 45 to 70, who reported on the same variables weekly over six weeks during the Israel-Hamas war in early 2025. Our findings inform theoretical models aimed to understand how extreme adversity and disasters affects older adults’ experience of aging.

